# A rare case of synovial sarcoma presenting as abdominal pain

**DOI:** 10.1002/ccr3.3432

**Published:** 2020-11-04

**Authors:** Kelsey Pan, Nida Waheed, James M. Smith, Zareen Zaidi

**Affiliations:** ^1^ Department of Internal Medicine University of Florida Gainesville FL USA

**Keywords:** abdominal pain, oncology, sarcoma

## Abstract

Abdominal pain can arise from numerous sources, including those extra‐abdominal. It is important to obtain additional imaging in the setting of clinical suspicion for malignancy.

## Graphical abstract

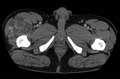

## 1 BACKGROUND

Sarcomas are a rare but aggressive type of malignancy that are often diagnosed late. This case describes an atypical presentation of sarcoma manifesting as abdominal pain, which previously has not been found in literature. We highlight the importance of pursuing additional workup when clinical suspicion for malignancy arises.

This case illustrates a diagnosis of synovial sarcoma of the thigh presenting as generalized abdominal pain. Synovial sarcoma is a malignant mesenchymal neoplasm that makes up 5‐10 percent of soft tissue sarcomas, and it tends to affect young adults.^1^ The neoplasm can arise from any soft tissue site, though it is most commonly known to arise from the lower extremities.^1^ Synovial sarcoma can often present aggressively. Delay in diagnosis is very common in synovial sarcoma. In fact, the time between the presence of a noticeable tumor and surgery is estimated to be between 2 and 4 years. In synovial sarcoma, it is common to have pain at the tumor site, often mistaken for a pulled muscle or ache, prior to the development of a clinically significant bulge.

Most cases of sarcoma present as localized pain and bulge at the affected site. No previous cases have described sarcoma manifesting as abdominal pain. This case integrates anatomy of abdominal and quadricep muscles with the understanding of abdominal pain. Specifically, we hypothesize that this patient's abdominal pain arises from the external compression of abdominal muscle insertion sites, along with nerve irritation causing referred pain along the abdominal sheath. This case demonstrates the necessity of obtaining additional imaging in the setting of weight loss and early satiety, especially when workup is initially unrevealing.

## 2 CASE PRESENTATION

A 47‐year‐old male triathlon athlete presents to primary care clinic with 2 months of abdominal discomfort that evolved from an epigastric to a generalized abdominal pain. It is associated with nausea, decreased oral intake, early satiety, odynophagia, and unintended weight loss of 20 pounds over 2 months. The pain is worse with meals and did not improve with the use of H2 blockers. He did not have unexplained fever, respiratory symptoms, or chest pain. He also had no changes in bowel movement, melena, or hematochezia. Review of systems was unremarkable other than feeling like he pulled a muscle in this right thigh a few months ago as he was training for a triathlon, for which he takes ibuprofen. His right hip and thigh area gradually grew more sore, contributing to performance decline. On examination, he had abdominal tenderness to palpation in all quadrants without organomegaly, guarding, rebound, or referred pain.

## 3 INVESTIGATIONS

Initial labs with CBC, CMP, and lipase were within normal limits. Patient underwent an upper endoscopy after initial presentation of abdominal pain associated with food intake and weight loss due to concern for upper GI malignancy. The EGD was negative.

**FIGURE 1:**
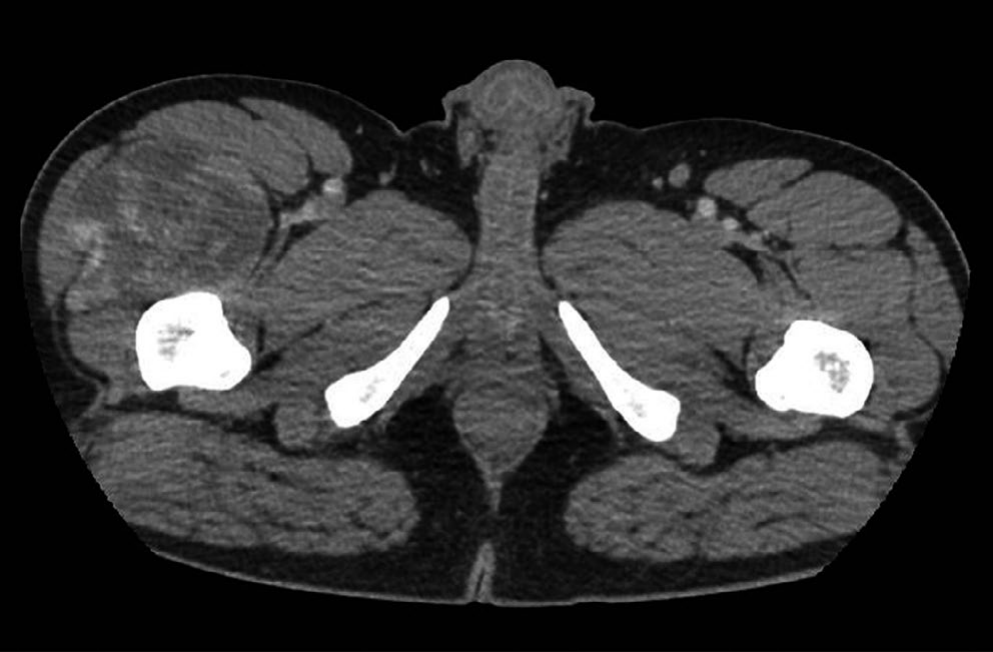
CT Abdomen/pelvis demonstrating 7.3 × 8.3 cm soft tissue mass with mass effect

**FIGURE 2:**
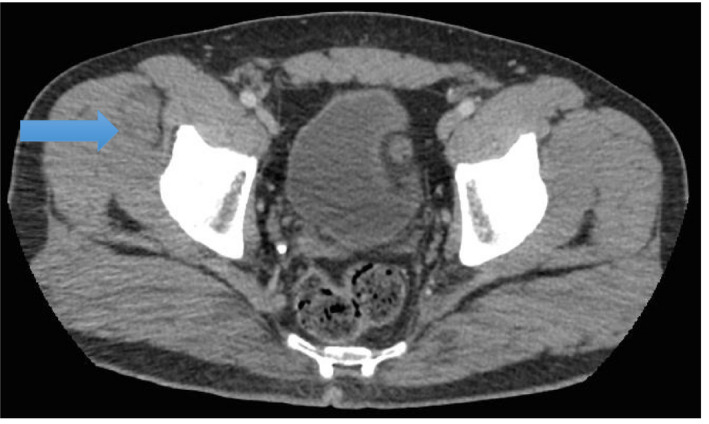
CT Abdomen/Pelvis showing soft tissue mass involvement at the level of the abdomen

In the setting of progressive symptoms, imaging with CT abdomen and pelvis was ordered. It showed a large soft tissue mass measuring 7.3 × 8.3 cm in the right anterior quadriceps femoris muscle, with mass effect upon the vastus lateralis, vastus medialis, and rectus femoris muscles (Figures 1 and 2).

Pathology from the mass biopsy revealed high‐grade monomorphic synovial sarcoma, spindle cell type. The synovial sarcoma had a SS18 gene locus rearrangement present.

## 4 DIFFERENTIAL DIAGNOSIS

There is an extremely wide range of possible causes of generalized abdominal pain, such as constipation, gastritis, gastroenteritis, irritable bowel syndrome, inflammatory bowel disease, malignancy, mesenteric ischemia, and more. However, this patient's unintentional 20 lb weight loss raised concern for malignancy. Basic labs and lipase were ordered to evaluate for pancreatitis. Upper endoscopy was ordered to evaluate intra‐abdominal causes of pain and weight loss, such as gastric ulcers or gastric malignancy. With unrevealing workup thus far yet ongoing symptoms, we next investigated with a CT abdomen and pelvis. Fortunately, the incidental CT finding of thigh mass helped expedite the workup that led to the patient's diagnosis of synovial sarcoma.

## 5 TREATMENT

He was treated with four cycles of neoadjuvant chemotherapy with ifosfamide and mesna, as well as neoadjuvant radiation therapy. He underwent surgery of the thigh mass 3 weeks after completion of neoadjuvant treatment. He continued on adjuvant chemotherapy with adriamycin and ifosfamide and has completed six cycles.

## 6 OUTCOME AND FOLLOW‐UP

The patient tolerated the chemotherapy and surgery well and had resolution of his abdominal pain after mass resection. Surgery revealed clean margins and no involvement of regional lymph nodes. CT chest revealed no pulmonary metastasis, making his stage T3N0M0. He has completed adjuvant chemotherapy and continues to participate in a physical therapy rehabilitation program. Unfortunately, in July 2020, he developed a new 1.3 cm pulmonary nodule in the left upper lobe concerning for metastatic disease.

## 7 DISCUSSION

There are numerous intra‐abdominal and extra‐abdominal pathologies that could manifest as abdominal pain. Extra‐abdominal causes of abdominal pain include pyelonephritis, ureterolithiasis, inferior wall cardiac ischemia, lower lobe pneumonia, tuberculosis, thyrotoxicosis, portal vein thrombosis, porphyria, and lymphoma.^2^ Though there are no prior cases in literature of extra‐abdominal malignancy presenting as abdominal pain, there have been reports suggesting mass effect causing abdominal pain. For example, Cho et al. describes a rare case of presacral myelolipoma causing abdominal pain through compression of nearby structures.^3^ Periaortic masses seen in retroperitoneal fibrosis have also been shown to cause abdominal pain through mass effect and fibroinflammatory infiltration.^4^ We suspect the patient's abdominal pain in our case of synovial sarcoma to arise from a combination of compression of local abdominal muscles and structures by the mass, in addition to neuropathic pain from surrounding nerve irritation.

Synovial sarcoma is a high‐grade soft tissue sarcoma that comprises 5 to 10 percent of all soft‐tissue sarcomas. It is commonly diagnosed during the early decades of life, though some cases have also occurred in adults in their eighth and ninth decades.^5^ Approximately 90% of cases occur before the age of 60 years.^6^ Synovial sarcomas often arise in the extremities as a localized pain at its origin even before the development of a noticeable bulge. The most common anatomical site effected is the extremities, especially the proximal to mid‐lower extremities.^1^ Synovial sarcoma can also affect the head and neck region, thorax, lumbar spine, and rarely the abdomen and pelvis.

Radiologically, synovial sarcomas tend to be large, lobulated, and frequently close to joints. For initial staging, magnetic resonance imaging is the imaging of choice due to its intrinsic signal characteristics and its superior ability to assess for tumor invasion.^7^ Positron emission tomography/ computed tomography (PET/CT) scan or CT of the chest, abdomen, and pelvis should also be performed for initial assessment and tumor surveillance.^8^ Diagnosis is made by biopsy. Commonly, synovial sarcomas contain the SS18 gene locus rearrangement, which is a characteristic translocation (X;18;p11;q11).^9^

There are two major histological subtypes of synovial sarcoma, biphasic and monophasic spindle cell types. There are also rarer subtypes including monophasic epithelial, poorly differentiated, calcifying and myxoid types. Histological features associated with a poorer outcome include mitotic indices of greater than 10 mitotic figures/10 high power fields, high nuclear grade, presence of rhabdoid cells, poor differentiation, and the presence of extensive tumor necrosis. On the other hand, extensively calcified synovial sarcoma is associated with a more favorable prognosis of 80% 5‐year survival.^1^

The patient described in our case had high‐grade, monomorphic synovial sarcoma, which signifies a relatively poor prognosis. Monomorphic synovial sarcoma has shown to have significantly worse overall survival (OS) than biphasic synovial sarcoma (42.3 vs. 59.7% 10‐year OS [*P* = .0111]). Higher histological grades have also been associated with worse prognosis, with grade 3 tumors having a 42.8% OS at 10 years, compared to 49.5% for grade 2 (*P* = .083).^10^ Prognostic value of the SS18 fusion gene, as seen in our patient, has been controversial in literature. However, a meta‐analysis by Kubo et al. suggests no significant difference in OS in patients with SS18‐SSX1 and SS18‐SSX2 fusion type, but that SS18‐SSX1 has been associated with worse progression‐free survival (PFS).^11^

Treatment for nonmetastatic sarcoma usually includes wide surgical excision, followed by radiation and sometimes adjuvant chemotherapy. Synovial sarcoma has shown to be more sensitive to chemotherapy compared to other adult soft tissue sarcomas, especially to alkylating agents. Therefore, first‐line chemotherapy includes ifosfamide and doxorubicin. It is estimated that 50% of patients with synovial sarcoma attain partial or complete remission with regimens containing ifosfamide and doxorubicin. Due to the rapid spread of disease and late‐stage diagnosis in the majority of cases, prognosis is poor. However, early recognition and diagnosis can significantly improve outcomes. Typically, 5‐ and 10‐year survival rates are about 60% and 50%, respectively. Metastatic disease occurs in about half of the patients, more commonly among adult patients. Common sites of metastasis include the lungs and pleura, bones, and lymph nodes.^1^ Poor prognostic factors include large tumor size, high histologic grade, older age at diagnosis, and vascular or osseous involvement.^8^, ^12^

## CONFLICT OF INTEREST

None declared.

## AUTHOR CONTRIBUTIONS

KP, NW, JS, and ZZ: designed the study, acquired and analyzed the data, wrote and revised the manuscript, gave final approval of the manuscript, and agreed to be accountable for all aspects of work.

## References

[1] Thway K , Fisher C . Synovial sarcoma: defining features and diagnostic evolution. Ann Diagn Pathol [Internet]. 2014;18(6):369‐380. 10.1016/j.anndiagpath.2014.09.002 25438927

[2] Fields JM , Dean AJ . Systemic causes of abdominal pain. Emerg Med Clin North Am. 2011;29(2):195‐210. 21515176 10.1016/j.emc.2011.01.011

[3] Cho MH , Mandaliya R , Liang J , Patel M . A case report of symptomatic presacral myelolipoma. Medicine. 2018;97(15):e0337. 29642172 10.1097/MD.0000000000010337 PMC5908593

[4] Tzou M , Gazeley DJ , Mason PJ . Retroperitoneal fibrosis. Vasc Med. 2014;19(5):407‐414. 25161213 10.1177/1358863X14546160

[5] Przybyl J , Sciot R , Rutkowski P , et al. Recurrent and novel SS18‐SSX fusion transcripts in synovial sarcoma: description of three new cases. Tumor Biol [Internet]. 2012;33(6):2245‐2253. 10.1007/s13277-012-0486-0 PMC3501176 22976541

[6] Fisher C . Synovial sarcoma. Ann Diagn Pathol [Internet]. 1998;2(6):401‐421. 10.1016/s1092-9134(98)80042-7 9930576

[7] Bakri A , Shinagare AB , Krajewski KM , et al. Synovial sarcoma: imaging features of common and uncommon primary sites, metastatic patterns, and treatment response. Am J Roentgenol [Internet]. 2012;199(2):W208‐W215. 10.2214/AJR.11.8039 22826423

[8] Pan M , Merchant M . Risk factors including age, stage and anatomic location that impact the outcomes of patients with synovial sarcoma. Med Sci [Internet]. 2018;6(1):21. 10.3390/medsci6010021 PMC5872178 29509716

[9] Eilber FC , Dry SM . Diagnosis and management of synovial sarcoma. J Surg Oncol [Internet]. 2008;97(4):314‐320. 10.1002/jso.20974 18286474

[10] Bianchi G , Sambri A , Righi A , Dei Tos AP , Picci P , Donati D . Histology and grading are important prognostic factors in synovial sarcoma. Eur J Surg Oncol. 2017;43(9):1733‐1739. 28579008 10.1016/j.ejso.2017.05.020

[11] Kubo T , Shimose S , Fujimori J , Furuta T , Ochi M . Prognostic value of SS18–SSX fusion type in synovial sarcoma; systematic review and meta‐analysis. Springerplus. 2015;4(1):375. 26217552 10.1186/s40064-015-1168-3 PMC4514732

[12] Casal D , Ribeiro AI , Mafra M , et al. A 63‐year‐old woman presenting with a synovial sarcoma of the hand: a case report. J Med Case Rep [Internet]. 2012;6(1):385. 10.1186/1752-1947-6-385 PMC3514372 23148739

